# Providers’ Perspectives on the Implementation of Mandated Local Health Networks for Older People in Québec

**DOI:** 10.5334/ijic.3098

**Published:** 2018-04-18

**Authors:** Paul Wankah, Yves Couturier, Louise Belzile, Dominique Gagnon, Mylaine Breton

**Affiliations:** 1 Université de Sherbrooke, Centre de recherche Charles-Le Moyne – Saguenay–Lac-Saint-Jean sur les innovations en santé, CA; 2 Université de Québec en Abitibi-Témiscamingue, CA

**Keywords:** Integrated care, older people, implementation factors, providers

## Abstract

**Introduction:**

In many countries, integrated care has been implemented to improve the quality, efficiency and patient experience of services. Understanding how integrated care is adopted in different settings may give insights into where, how and why different components of the organisational design work. The aim of this article is to understand how and why integrated care for older people has been implemented in different contexts from the perspective of providers.

**Theory and methods:**

The study uses an innovative composite framework for the implementation of integrated care models, which posits that structural, organisational, provider, innovation and patient factors influence implementation along six dimensions of integration. A qualitative multiple case study was done of three cases in Québec using document analysis and semi-structured interviews of 28 providers. Descriptive comparisons and thematic analysis were performed.

**Results:**

Providers considered that structural (government policy) and organisational (mergers) factors highly influenced the implementation of organisational and functional dimensions of integration, at the detriment of clinical integration. Provider, innovation and patient factors mildly or moderately influenced the implementation of integration.

**Conclusion:**

Structural and organisational factors were necessary conditions for the implementation of administrative components of integration, with great variability in the implementation of some clinical components.

## Introduction

Healthcare systems in developed countries try to improve the quality, coherence and continuum of services to specific vulnerable populations with complex needs, while concurrently improving the cost-efficiency of services by implementing community-based integrated health and social care programs [1,2,3]. These programs generally involve inter-organisational and inter-professional cooperation [4], coordination [4] and management of inter-organisational interdependence [4] in delivering services to patient groups that suffer most from the fragmentation of health care systems, such as cancer patients [5], people with cognitive disorders [6], or frail older people [7]. In this context, over the past two decades several integrated care programs for older people have been put in place around the world, notably the Program of All-inclusive Care for the Elderly (PACE) [8] and Social Health Maintenance Organisation (SHMO) [9] in the United States, Coordination for Professional Care for the Elderly (COPA) [10] in France, SA Health Plus [11] in Australia, Comprehensive Home Option for Integrated Care of the Elderly (CHOICE) [12], and Program of Research to Integrate the Services for the Maintenance of Autonomy (PRISMA) [13,14] in Canada.

Several studies have demonstrated that the benefits of integrating care for older people in research settings include better patient satisfaction, better continuity of services, better care coordination, better quality of services, lower cost of services, less fragmentation of services, and better interdisciplinary collaborations [13,15,16,17,18,19,20]. Despite these benefits, however, routine use of integrated care models is far from optimal, and there is a big gap between the implementation of experimental projects and the implementation of integrated care models in any given territory. As of now, there are no consistent guidelines on implementing integrated care models in everyday practice [21], and the still-significant gap between research and practice reduces the impact of integrated care models in real life settings. This gap may result from the complexities of implementing an integrated care program [21].

Current healthcare literature suggests that implementing an integrated care program involves a complex interaction of multiple actors, such as strategic actors (policymakers), tactical actors (managers), operational actors (health and social care providers), and users (patients and caregivers) [22] who may be located in different organisations (public organisations, community organisations, and private organisations) [22] within a healthcare system. Furthermore, the implementation of an integrated care program may be influenced by systemic factors [19] and characteristics of the innovation itself [19]. A better understanding of factors that influence the implementation of integrated care models for older people with complex socio-sanitary needs helps inform policymakers, managers, and providers regarding successful implementation, routinization and sustainability strategies. However, to date the factors that influence the implementation of integrated care models for older people have been infrequently studied or reported on in integrated care literature. Mackie and Darvil [19] systematically reviewed the literature on factors influencing the implementation of integrated health and social care for adults, reporting co-location of staff and teamwork, communication, integrated organisations, management support and leadership, resources and capacity, national policy, and information technology systems as the main enablers [20,23,24,25,26,27]. They concluded that there was limited evidence on factors influencing the sustainable implementation of integrated care and that more studies were needed to enhance the validity of the available evidence.

Health and social care providers, as operational actors, play an essential role in routinizing integrated care as they carry out their day-to-day routine tasks [22]. They deliver care to patients directly and assume important coordination roles such as case management. Organisational support for the innovation, participants’ attitudes to the innovation, and training activities have all been reported as factors that influence the implementation and routinisation of integrated care by providers [28,29]. The purpose of this study is to understand the implementation of integrated care models for older people as perceived by providers. Factors that influence the implementation of an innovation often have the potential to be either facilitating factors or barriers depending on the circumstances [24] (e.g. good leadership/poor leadership). Hence, in this article we use the term “influencing factors” rather than either “facilitating factor” or “barrier”.

This research sets two objectives:

1. To describe and compare the implementation of an integrated care model for older people in three different contexts according to the perspectives of providers.
2. To identify and understand factors that providers perceive as influencing the implementation of integrated care models for older people.

## Theoretical framework

The Rainbow Model of Integrated Care of Valentijn et al. [30] is a descriptive framework that distinguishes 59 items within six interlinked dimensions of integration: **clinical integration** (referring to clinical care coordination), **professional integration** (inter-professional coordination of services between various providers), **organisational integration** (inter-organisational coordination of services between various organisations), **systemic integration** (alignment of rules and policies within a system), **functional integration** (coordination of support systems) and **normative integration** (the extent of shared values and missions within the integrated system). This framework was used to generate a descriptive comparison [31] of the innovation along the lines of the six dimensions of integration.

The multilevel health innovations analysis model of Chaudoir et al. [32] is a renowned implementation science model that posits that five groups of factors influence the implementation of an organisational innovation. These include **structural factors** (factors linked to the external setting, such as the socio-economic and policy context in which the innovation is implemented), **organisational factors** (factors linked to the organisation where the innovation is being implemented such as organisational leadership), **provider factors** (factors linked to the providers implementing the innovation such as their willingness to change), **innovation factors** (factors linked to the innovation being implemented such as its complexity), and **patient factors** (factors linked to patients involved in the innovation, such as their health related beliefs).

**Figure 1:**
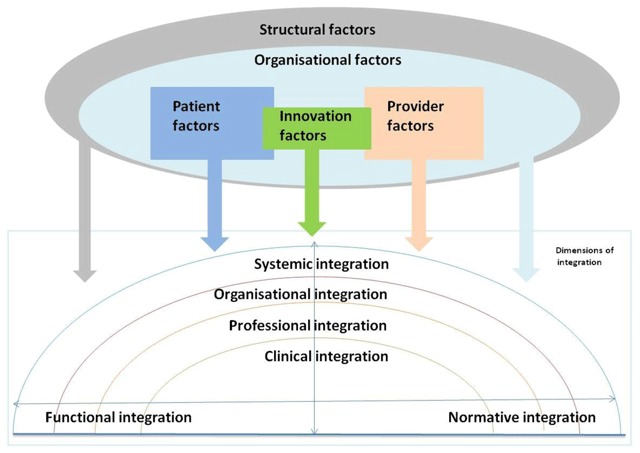
Composite conceptual model (combining the models of Valentijn et al. and Chaudoir et al.).

Figure 1 illustrates the theoretical basis of this research. The composite framework for the implementation of integrated care networks posits that five groups of factors (structural, organisational, innovation, provider, and patient) influence the implementation of six dimensions of integration (clinical, professional, organisational, systemic, functional and normative) of an integrated care model for older people. This study’s descriptive comparison and explanatory analysis is based on this framework.

## Context of the study

Québec is the second most populous province of Canada with a population of about 8.3 million people [33]. It has a publicly administered tax-funded health insurance system, ensuring universal medical coverage. The ministry of health and social services allocates block funding to health institutions such as hospitals and community health centres.

During the organisational reforms of the Québec health system in 2004, 95 Health and Social Services Centres were created, through the merger of public organisations (local community health centres, long-term care facilities, and some hospitals) [34]. The administrative merger of public organisations was the main mechanism the government of Québec chose to promote integration of health and social services [34,35,36]. These Health and Social Services Centres were mandated by government to create Local Health Networks through formal and informal inter-organisational agreements [37,38]. Various Local Health Networks addressing the needs of different sub groups of the population living on their territories were developed such as the Local Health Networks for Older People [39]. Its main components consists of: i) a joint governing board, ii) case management, iii) a Multiclientele Assessment Tool, iv) an individualised service plan, v) a health information system, vi) a common access point, vii) a family physician involved in the continuum of care for the older person, viii) an accessible geriatric team, and ix) an administrator responsible for the integrated care organisation [14,40]. Some of these components such as case management (clinical integration), a Multiclientele Assessment Tool (professional integration), the individualised service plans (clinical integration), information system (functional integration), and joint governance (organisational integration) represent items of the Rainbow Model of Integrated Care framework [30].

## Methodology

### Study design

Qualitative research methods are most suited to studying the experiences of participants while including their perspectives on social matters in a given context [41]. Holistic multiple case study is a qualitative methodological approach that facilitates the in-depth study of a situation in multiple units through multiple data sources [41]. This study is part of a wider international project, the iCOACH (Implementing Community-based models of care for Older Adults with Complex Health and social needs) project, carried out by a team of researchers from Québec, Ontario, and New Zealand [42]. This manuscript focuses on the perspective of providers regarding the implementation of a Local Health Network for Older People in three different contexts of Québec.

### Settings and participants

This project studies the implementation of Local Health Networks for Older People in three cases in Québec, representing three settings: highly urban (C 1), urban (C 2), and semi-urban (C 3). These cases were not selected to represent wider practices in Québec, but rather because they offered insights into implementing models of integrated care in their respective contexts [43]. In fact, one may consider that the organisation of health services differs depending on the level of the urbanisation of the setting. The three cases differ in terms of population density, geographic settings, and number of healthcare organisations [44]. Table 1 describes some characteristics of the different cases.

**Table 1:** Characteristics of the three cases studied.

Description	Case 1	Case 2	Case 3

**Geographical setting**	Highly urban area	Urban area	Semi-urban area (urban zones and rural zones)
**Total population**	421,342	164,666	41,927
**Surface area**	282 km^2^	325 km^2^	5,964 km^2^
**Population density**	1,494 people/km^2^	466.5 people/km^2^	7 people/km^2^
**Historical context**		Historical pilot site for a project on the integration of care for older people in Québec.	

Research participants included various providers (11 social workers, 10 nurses, 3 physicians, 2 occupational therapists, 1 community organiser, and 1 psychoeducator) who had worked for at least three years in their respective Local Health Network for Older People. Only providers who directly delivered care to patients were included in the study; providers with administrative or support clinical roles were excluded from the sample. Participants were purposefully selected based on their disciplinary training (e.g. physicians, nurses, social workers, occupational therapists) and where they worked in the Local Health Network for Older People (e.g. hospitals, health and social service centres, community organisations), to generate an adequate description of each case and identify a variety of factors influencing the implementation of Local Health Network for Older People from different perspectives.

### Data collection

Twenty-eight semi-structured face-to-face interviews, ranging from 50 to 90 minutes, were conducted by the researchers between May 2015 and December 2016. Each participant was provided with information about the aims, procedures, and ethical aspects of the study. An informed consent form was signed by each participant before the interview. Data saturation was deemed to have been achieved when the last three interviews added no new information, after which no further providers were invited for interview [45]. All interviews were audio recorded and transcribed verbatim.

Researchers from the Québec branch of the iCOACH project collaboratively developed a topic list for interviewing providers. The six main themes covered were: 1) Organization of care for older people living with complex social and health needs; 2) Links to community resources; 3) Self-management support; 4) Innovation and evidence to support care (also known as clinical decision support); 5) Health information systems they used; and 6) Organizational approach and culture in the change of care. Piloting indicated that the interview structure and questions were clear and suitable for the purposes of the study. Each interview started with an open-ended question that encouraged interviewees to talk freely about their experiences, thereby reporting what they perceived as being most important. Subsequently, they were prompted with more specific questions to help clarify their answers.

Other data sources included yearly government reports on the monitoring of Local Health Networks for Older People. We searched for specific information from specialised websites, such as the Statistics Canada website.

### Data analysis

Qualitative data analysis was done using the NVivo 11 software package [46]. The Québec research team refined the iCOACH projects’ provider codebook, adapting the codes and themes to better match the Québec data. Then two researchers (WP and LB) independently coded the first three interviews, after which they met, compared codes for providers’ perspectives, and discussed any differences. The coding of the next three interviews showed a significant overlap in the coding of both researchers, with an inter-judge reliability greater than 80% [47,48]. Thereafter, one researcher coded the remaining interviews.

For the descriptive comparison [31] of the three cases, the Rainbow Model of Integrated Care framework was used as a codebook to organise the data. WP initially made an extensive and detailed descriptive summary of each case. Subsequently, a highly synthesized and simplified descriptive summary of each case was prepared, which facilitated the comparison of the three cases according to the six dimensions and 59 items of the Rainbow Model of Integrated Care [30] descriptive framework (Figure 1). Next, MB, LB, YC and DG both reviewed the detailed and simplified descriptive summaries. Differences in opinions were discussed and the final descriptive comparison of the three cases was reached by consensus.

The thematic analysis technique [31,49] was used for the identification and analysis of factors influencing the implementation of the Local Health Network for Older People. This was done in two steps. First, the Multilevel Health Innovations Analysis Model was used to create a codebook which facilitated the identification of factors that providers perceived as positively or negatively influencing the implementation of the Local Health Network for Older People. Then the dimensions of integrated care that were most likely to be influenced by each factor were identified by induction and organised on the Rainbow Model of Integrated Care. Next, the team reviewed the themes of factors influencing the implementation of each dimension of a Local Health Network for Older People, and together agreed on the degree of influence of each, expressed on a three-point scale (mild, moderate, or high), according to the providers’ perspective and documentary analysis. Differences in opinions were discussed so that the final thematic analysis of the three cases was reached by consensus.

The quotes used to illustrate the results have been translated from French to English for the purpose of this article.

### Ethics

This study was approved by the Research Ethics Committee of Charles-Le Moyne Hospital (ref. number CE-HCLM-15-001).

## Results

### How the Local Health Network for Older People was implemented

Descriptive comparison of the implementation of each of the six dimensions of integration are presented in table form under each of the following sections, with similar cross-case perspectives in a merged row and differing perspectives in a fragmented row.

#### The clinical dimension of integration

The clinical dimension consists of 12 items. Table 2 presents the similarities and differences in their implementation. The “case management” and “population needs” items differed across the three cases. Three different models of care coordination through “case management” were implemented in each case.

**Table 2:** Providers’ perspectives on the implementation of the clinical dimension of the Local Health Network for Older People.

Component	Case 1	Case 2	Case 3

**1) Centrality of client needs**	– Focused on the physical, mental, and social aspects of users’ health as indicated by the Multiclientele Assessment Tool. – Difficulties balancing patients’ needs to the services offered.		
**2) Case management**	– Only social workers were “case managers”. – Only nurses are “main providers”.	Any provider (nurse, social worker, occupational therapist) could be a “case manager”.	Social workers or nurses could be “main providers”.
**3) Patient education**	– Educating patients as part of their informed consent and shared decision-making activities.		
**4) Client satisfaction**	– Providing care for client’s needs with the resources available. – There are no formal mechanisms to measure the client’s satisfaction.		
**5) Continuity**	– Fragmented care still existed in the Local Health Networks, though mechanisms were put in place to facilitate care continuity.		
**6) Interaction between professional and client**	– Patient engagement during shared-decision making and consent. – Administrative and legal obligations such as explaining their roles or duties, sometimes hindered interactions.		
**7) Individual multidisciplinary care plan**	– Creating multidisciplinary individualised care plans for patients. – Variable use of care plans within and across the cases.		
**8) Information provision to clients**	– Variable criteria of access to certain local resources, (e.g. local clinics), sometimes they are not known.		
**9) Service characteristics**	– Services were provided, depending on the individual patient’s needs and the capacity of the Local Health Network. – There were patient waiting lists for some services.		
**10) Client participation**	– Endeavouring to engage all patients in shared decision making, though this was not always possible in practice.		
**11) Population needs**	– Providers were more focused on the needs of the patients than those of the wider population.		
	Interpretation services were offered for the multicultural population.	Some community organisations offered relief services for exhausted caregivers.	Transport services were needed for patients in rural zones of the territory.
**12) Self-management**	– Self-management support that aimed at increasing the autonomy of patients were mostly information on local community services.		

Regarding provider-patient interactions, all providers reported sharing decision-making with patients and their caregivers. This facilitated patients’ participation in the elaboration and implementation of care plans, transmission of information to the patient, prioritisation of the needs patients felt most strongly about, and self-management support.

“I tell them [patients] all the time, I’m here for you, to present you the situation, I show you what I know about the services, and you make the decisions.” (Nurse C2)

Though fragmented care still existed in the Local Health Networks, mechanisms such as liaison nurses and inter-organisational referral forms (the *Demande de Services Inter Etablissements* – DSIE) ensured temporal continuity of services.

#### The professional dimension of integration

The professional dimension consists of 11 items. Table 3 presents the similarities and differences in their implementation. The three cases differed in the mechanisms used for performance management, and focused inter-professional education was only reported in Case 1.

**Table 3:** Providers’ perspectives on the implementation of the professional dimension of the Local Health Network for Older People.

Component	Case 1	Case 2	Case 3

**13) Inter-professional education**	– Continuous education and inter-professional team work.		
	Focused inter-professional training activities in the X pilot project.		
**14) Shared vision between professionals**	– Multidisciplinary teams developed the content of care. – Variable and unequal hierarchical team work dynamics, in favour of health care organisations over the other partners.		
**15) Agreements on interdisciplinary collaboration**	– No formal agreement on interdisciplinary collaboration was mentioned. – Shared clinical tools may foster interdisciplinary collaborations. – Heavy workloads discourage interdisciplinary collaborations.		
**16) Multidisciplinary guidelines and protocols**	– Same government-issued planning tool and Multiclientele Assessment Tool (OEMC, *Outil d’Évaluation Multiclientèle*) in Québec. – Used by different groups of providers (e.g. nurses, social workers, occupational therapists).		
**17) Inter-professional governance**	– Governance structure consisting of health and social care providers who are jointly accountable for services delivered to patients.		
**18) Interpersonal characteristics**	– Equality, trust, and respect between the different partners in a multidisciplinary team. – Previous successful collaboration experiences facilitated current collaborations.		
**19) Clinical leadership**	– No provider stood out as a champion in the implementation of this Local Health Network.		
**20) Environmental awareness**	– Rarely referring to the socio-economic and political climate of their Local Health Network, they seemed to endure the reforms, instead of participating in them.		
**21) Value creation for the professional**	– Capacity-building through regular interdisciplinary collaborations greatly depended on individual providers.		
**22) Performance management**	– Mix-up between organisation performance (defined by management goals and activities volume) and clinical performance (defined by service quality goals). Only organisational performance is considered.		
	Performance indicators were presented on a monthly basis by team leaders.	Performance was measured based on the activities of the providers, such as the number of completed evaluations.	Performance was measured every three months by team leaders.
**23) Creating interdependence between professionals**	– Lack of knowledge of activities and situations of other providers usually led to fragmented professional care, though providers sometimes developed interdependent approaches in care delivery (e.g. nurses and nursing assistants).		

All professionals reported using the same government-issued Multiclientele Assessment Tool (OEMC, *Outil d’Évaluation Multiclientèle*), which facilitated inter-professional collaboration.

“I evaluated the patient, and then I had a meeting with the case manager where I filled my part of the OEMC pertaining to his [patient’s] transfer and all that.” (Occupational therapist C2)

In line with multidisciplinary guidelines and protocols, case managers and “main providers” regularly organised multidisciplinary meetings to discuss the needs of complex patients. Inter-professional collaborations were perceived as adding value to the individual providers, creating interdependence, and contributing to reinforcing their shared vision.

#### The organisational dimension of integration

The organisational dimension consists of 13 items. Table 4 presents the similarities and differences in their implementation.

**Table 4:** Providers’ perspectives on the implementation of the organisational dimension of the Local Health Network for Older People.

Component	Case 1	Case 2	Case 3

**24) Value creation for organisation**	– Partners from the community and private sectors provided complimentary services to the public organisations. – Some issues regarding the coherence and management of services delivered by the different partners.		
**25) Inter-organisational governance**	– Not assessed.		
**26) Informal managerial network**	– Not assessed. (Providers had limited knowledge of the governance of their organisations. Few mechanisms to participate in the governance of their organisations.)		
**27) Interest management**	– Favourable organisational climate for the combined interests of the strategic, tactical, and operational levels. – Deplored the frequency and magnitude of changes in their Local Health Networks. – Local community organisations are financially dependent on their public partners, and this may influence their missions and the services they deliver.		
**28) Performance management**	– Few strategies to distribute the workload so as to reach management targets over the fiscal year.		
**29) Population needs as binding agent**	– Inter-organisational collaboration mildly considered the needs of the population. They were more focused on managerial targets of individual organisations, such as reducing waiting lists.		
**30) Organisational features**	Mega-urban Health and Social Services Centre characterised by high population density, multiple organisations, and proximity of specialised services.	Urban Health and Social Services Centre characterised by moderate population density, sufficient number of organisations, and proximity of specialised services.	Semi-urban Health and Social Services Centre characterised by a low population density on a large territory, limited number of organisations, and sparse specialised services.
**31) Inter-organisational strategy**	– The Local Health Network was organised around the Health and Social Services Centre, which arranges the sharing of some resources (financial, material and human) with its partners. –Two main strategies; administrative mergers of some public organisations, and linking strategies of various partners.		
**32) Managerial leadership**	– Centralisation of decision making powers to the ministry of health and social services.		
**33) Learning organisations**	– Not assessed.		
**34) Location policy**	– Several co-location strategies amongst partner organisations, for example the merger of partner organisations, were thought to be beneficial to their partnerships.		
**35) Competency management**	– Not assessed.		
**36) Creating interdependence between organisations**	– Organisational interdependence occurred through shared responsibility for delivery of care to clients, coordinated by the Health and Social Services Centres.		

The integrated care network was organised around the Health and Social Services Center which established partnerships with organisations on its territory. The number of partnerships differed across the three contexts, with the mega-urban case (C1) establishing more partnerships than the urban and semi-urban cases.

“Our patients are placed in those four [private] long-term care centers…We are overwhelmed in our CHSLDs [public long-term care centers], there is no room. We purchased some beds….162 beds in the four [private] long-term care centers.” (Nurse C1)

Partner organisations mostly provided complementary services, which eventually led to a sort of organisational interdependence. The perspectives on organisational features and learning organisations differed in the three cases.

Notably, providers did not have much information on managerial-level items such as inter-organisational governance, or competency management.

#### The systemic dimension of integration

The systemic dimension consists of six items. Table 5 presents the similarities and differences in their implementation.

**Table 5:** Providers’ perspectives on the implementation of the systemic dimension of the Local Health Network for Older People.

Component	Case 1	Case 2	Case 3

**37) Social value creation**	– Major structural reforms led to administrative integration that was less felt at the clinical level.		
**38) Available resources**	No major lack of resources.		Lack of sufficient human resources.
**39) Population features**	– More concerned by the features of their clients than those of the population.		
	Substantial immigrant population with cultural specificities.	Many isolated older people with poor social networks.	Many older people dispersed over a large territory.
**40) Stakeholder management**	– Centralisation of decision making powers which created a distance between management and providers. – The Health and Social Services Centre was mandated by government to establish and coordinate partnerships with local community organisations and private organisations.		
**41) Good governance**	Not assessed.		
**42) Environmental climate**	– The three cases shared the same socio-economic and political climate marked by marked by successive health system reforms, raising concerns regarding the benefits of these reforms at the clinical level.		

All providers shared the same socio-economic and political climate marked by frequent organisational reforms mainly championed by the government. The semi-urban case (Case 3) lacked human resources, and it also had an ageing population that was sparsely distributed over a large rural territory.

“Of course, from time to time we also have difficulty recruiting people to provide services… it’s more difficult, I think, in rural areas.” (Social worker C3)

Providers did not have much information on the governance level-item.

#### The functional dimension of integration

The functional dimension consists of six items. Table 6 presents the similarities and differences in their implementation. Feedback mechanisms varied across the three cases.

**Table 6:** Providers’ perspectives on the implementation of the functional dimension of the Local Health Network for Older People.

Component	Case 1	Case 2	Case 3

**43) Human resource management**	– Administrative mergers facilitate human resources management, but have little impact on the work climate. – Staff of partner organisations often collaborate in care delivery.		
**44) Information management**	– Multiple unaligned health information systems at the operational level.		
**45) Resource management**	– Resources do not always meet the needs of clients. – Funding models focus on the volume of services offered, and do not take into consideration the quality of care.		
**46) Support systems and services**	Not assessed.		
**47) Service management**	– There is coordinated 24-hour assistance for users and providers, facilitated by a unique telephone number and a shared point of access for the Local Health Network. – Various regulations complexify the use of these resources.		
**48) Regular feedback of performance indicators**	Providers were given feedback during monthly meetings with their managers.	None mentioned.	Feedbacks reflected volume of services.

Providers reported having multiple computerised information systems that were mostly unaligned, and this hindered information continuity.

“So they [nurses] have access to my computerized patient notes, […] while I do not have access to the details of their [patient] notes, […] but given my role as a case manager, it’s a big barrier.” (Social worker C2)

The staff of partner organisations generally worked together in the community, and resources were mostly managed by the Health and Social Services Centre. Only Case 1 reported any feedback of performance indicators.

Providers did not have much information on support systems and services.

#### The normative dimension of integration

The normative dimension consists of 11 items. Table 7 presents the similarities and differences in their implementation. No difference was found across the three cases.

**Table 7:** Providers’ perspectives on the implementation of the normative dimension of the Local Health Network for Older People.

Component	Case 1	Case 2	Case 3

**49) Collective attitude**	– Providers are overwhelmed by the frequent structural reforms and their individual workloads.		
**50) Sense of urgency**	– Providers did not understand the need for frequent organisational changes. – They had limited knowledge of the concept of integrated care.		
**51) Reliable behaviour**	– The pertinence of the innovation is lost with the high turnover of providers and managers.		
**52) Conflict management**	– Not assessed.		
**53) Visionary leadership**	– Not assessed.		
**54) Shared vision**	– The main aim of the Local Health Network was to maintain older people with complex needs at home with quality care for as long as possible with the resources available.		
**55) Quality features of the informal collaboration**	– Inter-professional collaborations were mostly satisfactory. It seemed to benefit teamwork. – Managerial-professional collaborations were less satisfactory.		
**56) Linking cultures**	– Administrative mergers did not change the cultures of the various health organisations.		
**57) Reputation**	– Not assessed.		
**58) Transcending domain perceptions**	– Not assessed.		
**59) Trust**	– Trusting relationships between providers and managers facilitated teamwork.		

The benefits of maintaining complex patients at home with quality care for as long as possible with available resources was clearly understood by all providers, who reported shared values and satisfactory inter-professional collaborations. On the other hand, provider–manager collaboration was not always smooth.

Several items of this dimension were not assessed by providers.

In summary, the descriptive comparison brought forth two main observations. First, despite the major reforms carried out by the government at the systemic level of integration, there was significant overlap in how the Local Health Network for Older People were implemented in the three cases studied. Second, providers were able to discuss most items of the clinical, professional and functional dimensions of integration adequately, but often had very limited information on items in the organisational, systemic, or normative dimensions of integration. Both observations suggest that, despite the overall conceptual similarity in the implementation of integrated care in the three cases studied, providers generally had a limited knowledge of normative processes at the managerial and policy level of integration, which may reflect a certain loss of the primary purpose of the innovation.

### Factors which influenced the implementation of the six dimensions of the Local Health Network for Older People

#### Structural factors

Most providers reported that government policies and funding support for the Health and Social Services Centres greatly influenced implementation of the Local Health Network for Older People (Table 8). As one respondent expressed, articulated government support (laws and policies) could directly influence providers.

“According to Bill 90 (of 2002), it is us, the nurses, who have to put in place the [treatment] plan, the training for the staff and everything, so that the acts are carried out according to the standards of nursing care […] When it is a delegated act, according to Bill 90, it is the clinical nurse. So I go there continuously to train new staff.” (Nurse C1)

**Table 8:** Structural factors perceived as influencing the implementation of six dimensions of the Local Health Network for Older People.

Factors	Clinical dimension	Professional dimension	Organisational dimension	Systemic dimension	Functional dimension	Normative dimension

**Government policy and funding support**	+	+	+++	+++	+++	++
**Managers and policy makers use of innovation**		+	++	+++	++	++
**Characteristics of the population**		+	+			
**Geographical setting (urban zones vs rural zones)**		+	+			

Degree of influence: + mild influence; ++ moderate influence and +++ high influence.

Articulated government support could also influence providers indirectly. For instance, different laws and policies drove the mergers of public health organisations (leading to the creation of the Health and Social Services Centres), while at the organisational level government policies mandated and empowered the Health and Social Services Centres to create partnerships with organisations of their territories to deliver care to older people. Concrete government support in the form of budgetary, material and human resource allocations to the Health and Social Services Centre helped form inter-organisational links through mechanisms such as the provision of financial subsidies or personnel to partner organizations. Government policies and support also aided other components of the Local Health Network for Older People, as in the case of the multidisciplinary evaluation tool, which facilitated professional and clinical integration.

On the other hand, more than a decade after the creation of Health and Social Services Centres, some components, such as case management, that were neither clearly defined at the ministerial level nor supported by the government, were poorly implemented in two cases.

“The procedures were … Well, in fact, they still are not clear. Great! You appoint me case manager, starting today, here are your pagers, but people are phoning … my role, the procedures are not yet available. So I think that’s a major shortcoming….” (Social worker C1)

Managers and policymakers regularly used data generated from the evaluation of patients with the multidisciplinary evaluation tool. Though most providers did not have any feedback on the usage of these statistics, some providers in Cases 1 and 3 indicated that their performance was partially measured with this data. This motivated them to regularly carry out patient evaluations with the multidisciplinary evaluation tool. This pressure to measure the volume of activity has detracted from the clinical dimension of the evaluation.

Some providers felt that they did not have much of a say in managerial issues in their Local Health Network.

“There is no interest, at present, for management, to seek out the knowledge of senior staff [providers]. In the past, during our clinical committee meetings, senior staff were always there to guide the younger ones. We are senior staff, the most… our expertise is no longer desired by the clinical committee, by the boss.” (Nurse C3)

In a broader sense, this may lead to a lack of provider engagement in the design, execution, or monitoring phases of Local Health Network for Older People implementation. This lack of engagement may explain why providers had limited knowledge of processes at the managerial and policy levels of implementation of their Local Health Network for Older People.

Characteristics of the population, such as the relative proportion of immigrants, also influenced Local Health Network for Older People implementation.

“There are different community organizations, but that’s because we have a lot of cultural communities […] with [different] challenges, because they are often communities that want to keep [their patients] at home for very, very, very long. And sometimes it can be difficult in terms of the resources that can be mobilised.” (Social worker C1)

#### Organisational factors

Most providers are unequivocal concerning the impact of mergers on their daily experience in their Local Health Networks (see Table 9). Some providers reported negative experiences.

“I am convinced that it has deteriorated. I started in [X] local community health centre when we were a very small team. So, when there was shortage of staff, well, we just crossed the corridor to meet the human resources manager, she would immediately find a replacement […]. We later merged with [Y] local community health centre. Well what we noticed, was that, strangely, we didn’t know the human resource manager anymore and all of a sudden there was no more equipment. Subsequently we merged with [Z] hospital, which was even worse. There, if you call in to have a replacement, well, the priority is the emergency room; it is not the home care unit so there is no one… no one to replace us. We don’t have equipment anymore, we often get calls informing us not to hospitalise patients, as there are no beds available in the hospital. So I don’t know, where did they all go to?” (Nurse C1)

**Table 9:** Organisational factors perceived as influencing the implementation of six dimensions of the Local Health Network for Older People.

Factors	Clinical dimension	Professional dimension	Organisational dimension	Systemic dimension	Functional dimension	Normative dimension

**Merging organisations**		+	+++	+++	++	+
**Networks and collaborations**	+	++	+++	+++	++	++
**Shared vision**		+	+++	+++	++	++
**Formal or informal strategies of communication**		+	+			
**Shared decision making**	++	+	+++			
**Engagement of providers by managers**			++			
**Health information system**	+	++	+++	+++	++	+

Degree of influence: + mild influence; ++ moderate influence and +++ high influence.

Specifically, the organisational mergers generated two main barriers: 1) the loss of physical proximity between providers and the managerial level, impeding transmission of information to the relevant manager, which inevitably resulted in neglect of the providers’ needs; 2) in the larger merged organisations, most of the human and material resources seemed to be directed towards the acute healthcare unit (hospital emergency services) at the expense of the other units. Hence, the large-scale managerial administrative structure undermines the project’s ability to integrate services within clinical practice, at least in the short term.

Meanwhile, the merged organisation (the Health and Social Services Centre) formed extensive networks and collaborations with partners. For example, in Case 1 the Health and Social Services Centre purchased 162 beds in four private residential facilities on its territory. Such networks and collaborations between partner organisations in the Local Health Network were perceived as enhancing the implementation of the Local Health Network for Older People by facilitating organisational, professional, and functional integration.

Providers shared the vision of maintaining patients at home for as long as possible in the best medical state possible. This reflected effective top-down communication of the vision held at the strategic and tactical levels of the Local Health Network which aimed to control healthcare costs while improving the quality of health services by caring for patients with complex needs in their homes. Hence, providers frequently reported having to advocate, through formal or informal means, on behalf of their patients at different points in the Local Health Network, especially when the patients’ clinical states were deteriorating. This shared vision fostered professional, organisational, systemic, functional, and normative integration.

Providers reported that shared decision-making with patients and caregivers was a central principle that was encouraged by their respective organisations, though it was sometimes difficult to implement.

“I explain to them what is happening and then I ask them, are there any other services you would like? There, I … I do it very openly. Then, when I they talk about, I check if it is possible or not, then I explain to them what is possible or not in reality.” (Nurse C2)

This enabled providers to actively engage users in the process of care delivery, informing them on their clinical state and services they would need, obtaining their consent, and providing self-management support to the client. This process facilitated the education of users and enhanced client satisfaction by addressing the needs they felt most strongly about. In the long run, providers established a trusting relationship with patients, thereby improving patient adhesion to care.

Some providers had positive experiences while using health information systems.

“Then in (X) health information system, we are able to see the path our colleague would take on a given day. You know, I click on the provider, and then I want to know, for example, the nurse in the other area, where, how many patients she has, then where she goes, if she goes in the neighbourhood of … of one of my patients. Can she visit one of my patients?” (Nurse C1)

Nurses in Case 1 reported that electronic health information systems helped them coordinate care with their colleagues, maintain relational continuity of care with the patient, and organise their schedules/caseloads during their group meetings. Despite all of these benefits, several barriers were reported, namely, the existence of multiple unaligned health information systems, older versions of software that were difficult to use, and difficulties in transferring information between different software applications.

#### Provider factors

Most providers had a positive attitude towards several components of the Local Health Network for Older People and were therefore very likely to implement the innovation (Table 10). The continuity produced by the integrative model had an effect in terms of inter-professional collaboration.

“Interdisciplinarity, it is a plus for the patient […] And we … we draw up [individualized] care plans. So what makes us … we … we have lots of … software of … of … how do we call it? The evaluation grids, there, we became pros at that, so … we have so much material to be able to assess the situation well, to follow up on all this. Therefore, interdisciplinarity is … it is facilitated by all these work tools.” (Social worker C2).

**Table 10:** Provider factors perceived as influencing the implementation of six dimensions of the Local Health Network for Older People.

Factors	Clinical dimension	Professional dimension	Organisational dimension	Systemic dimension	Functional dimension	Normative dimension

**Attitude to the intervention**	+	+	+		+	++
**Multidisciplinary teams**		+++	++	++		+
**Personal attributes**	+	+	+		+	++
**Level of education**	+	++				
**Workloads**		++				
**Willingness to work in semi-urban zone**	+	+				

Degree of influence: + mild influence; ++ moderate influence and +++ high influence.

Providers were generally enthusiastic about, and felt empowered by, working together in multidisciplinary teams, which was further facilitated by the clinical tools provided by the innovation. Sometimes, difficulties in accessing physicians were perceived as a barrier to teamwork, but we also noted that physicians generally did not report difficulties in accessing other health professionals. In fact, the physicians seemed to appreciate the ready availability of the multidisciplinary teams, especially case managers (where they existed), who contributed substantially to maintaining continuity between the physician and the multidisciplinary team. Generally, providers reported that personal attributes such as openness, agreeability, and conscientiousness facilitated group work. All providers seemed to be generally satisfied with their team work.

In all three cases, the case manager function was assumed by providers from different disciplines, but with at least a relevant university degree. Providers’ level of education became a barrier to the implementation of the case management function in Case 3 since there was insufficient qualified personnel in that semi-urban setting. Some providers, especially those in the semi-urban setting, perceived their workloads as being too high. This may be related to the fact that the semi-urban zone was relatively understaffed as compared to the other settings, as providers were not very willing to work in semi-urban zones.

#### Innovation factors

Remarkably, the case management role was highly adaptable and trialable (Table 11). Adaptability was clear from the fact that in Case 1 only social workers were case managers, while in Case 2 all health providers could be case managers, and in Case 3 no case managers were deployed, for the reasons previously explained. Providers in Case 1 reported that different models of case management had been tried over the years. They had started off by having different types of providers (social workers, nurses, occupational therapists, etc.) as case managers, then switched to assigning a few social workers in the homecare unit as case managers, and finally settled on designating all social workers in the home care unit as case managers.

**Table 11:** Innovation factors perceived as influencing the implementation of six dimensions of the Local Health Network for Older People.

Factors	Clinical dimension	Professional dimension	Organisational dimension	Systemic dimension	Functional dimension	Normative dimension

**Adaptability**			++	++		
**Trialability**	+	+	+			
**Cumbersomeness**		+	+			
**Lengthy duration**	+					
**Complexity**	+	+				
**Flexibility of provider**		+	+			
**Applicability**	+					

Degree of influence: + mild influence; ++ moderate influence and +++ high influence.

Other components of the innovation, such as the multidisciplinary evaluation tool, were perceived as cumbersome.

“I often have social workers calling me from the hospital ‘Mrs. X is in the hospital and…. We wanted to check on her level of functional autonomy at home and the services she received.’ I sometimes think maybe they don’t have time to check the multidisciplinary assessment tool. I have often told them that they can check it on the health information system, you know… But it has 19 pages, the multidisciplinary assessment tool is not concise.” (Social worker C1)

Other providers reported that it took too much time to complete one multidisciplinary evaluation using the tool, even though the government required them to do at least one evaluation per year.

#### Patient factors

Providers reported that patient characteristics also influenced how they carry out their duties.

“Because it’s for sure that… for me, most people I meet have cognitive disorders. So, if I want to have a fair evaluation, I need to talk to the immediate entourage of the person.” (Social worker C3)

Providers also noted that, since it is much easier to work with patients and caregivers who receive support from their immediate and extended families, in some cases they would set up meetings with the patient’s family to discuss the needs of the patient and how the family can support the patient and caregiver. A family meeting is a strategy to simultaneously address the needs of the patient and to invite the support of family members. On the other hand, providers were reluctant to provide care to aggressive patients, especially at their homes, where the provider is in an unfamiliar environment.

Patient satisfaction was a very important factor, which motivated providers to involve patients as much as possible in decision-making related to their own care.

“The person… has to be satisfied with the care. But it is certain that one tries as much as possible with the person, has decision making.… But it’s really case by case.” (Physician C2)

Providers reported being more likely to provide care that was immediately beneficial to the patient.

## Discussion

The aims of this multiple case study were to describe and compare the implementation of an integrated care model for older people in three different contexts and to explore providers’ perspectives on factors influencing the implementation of such a model. The Rainbow Model of Integrated Care framework [30] facilitated a descriptive comparison along six dimensions of integration of the Local Health Network for Older People as perceived by the providers in the three cases studied.

Overall, the descriptive comparison revealed a high degree of similarities in the implementation of integrated care models across the three cases studied. This finding may reflect government’s influence in the top-down approach chosen to implement this integrated care model, and the profound structural changes they championed [34]. This is in line with contemporary literature on the implementation of mandated innovations, where the “mandator” may greatly influence the implementation of an innovation by creating enabling conditions [50]. On the other hand, the implementation of some items, less structural ones, differed across the cases (e.g. case management). Contemporary literature suggests that elements of the local context (availability of resources, organisational culture or the willingness to adopt new practices by local actors) may significantly shape the local implementation of innovations [51]. The most relevant aspects of the descriptive comparison of the six dimensions of integration are discussed in the next paragraphs.

In principle (e.g. ministerial policy), integrated care models aim at promoting the patient-centred care, a needs-oriented system of care delivery [35,52]. The clinical dimension of integration (Table 2) was marked by difficulties balancing patient needs with services offered, suggesting that though the policy document promotes a needs-oriented system, providers viewed the system as still being service-oriented. Though providers reported that they engaged users (patients/caregivers) in shared decision-making activities which improved provider-client interactions, and an understanding of their specific needs, they still faced challenges related to the limited resources available and fragmentation of services within and between different units of the integrated care model. This was compounded by the fact that the three cases studied had different care coordination models through case management. This could be attributed to the mandator’s (the government) lack of specific directives on the “case management” function in Québec [14].

Contemporary literature concurs that inter-professional collaboration is an essential component of integrated care models [53,54,55]. The government issued “Multiclientele Assessment Tool” (Table 3) was perceived as improving information sharing between various providers. Though the providers reported formal (e.g. pilot projects) and informal (ad hoc meetings) collaboration models, the team work dynamics weighed on the side of the medical needs at the expense of the social needs of the patient [34].

The most interesting results of the organisational dimension (Table 4) and normative dimension (Table 7) is what providers did not report. Little information was reported on four items of the organisational dimension (inter-organisational governance, informal managerial networks, learning organisations, and competency management) and four items of the normative dimension (conflict management, visionary leadership, reputation and transcending domains and perceptions). Basically, providers displayed knowledge gaps of managerial and governance level items. This raises the question of the lack of engagement of providers, or the disinterest of providers in the implementation process of the integrated care model [14]. Meanwhile, several studies suggest that engaging multiple stakeholders, including providers, in the design, implementation and monitoring of innovations may enhance their adoption [14,56,57]. The ‘natural bias’ concept, where frontline staff tend not to know much about structural, managerial and organisational issues in any organisation, may also explain these knowledge gaps [58]. Managerial perspectives may shed more light on these items.

The government of Québec created major structural reforms that promoted centralised governance as enabling conditions for the implementation of integrated care models (Table 5). These reforms were perceived as being better implemented at the administrative levels of the three cases, at the expense of the clinical level of integration. There were inherent systemic inequities in human resources allocation such as underserviced rural zones, and the population contexts of the three cases varied from a dense multicultural immigrant population in case 1 to a sparse older population in case 3. These systemic inequities and variable population contexts suggests that population needs should be taken into consideration in the local implementation of integrated care models [14,35]. Turgeon et al. [34] pointed out that frequent successive structural health system reforms, as is the case of Québec, do not allow enough time for frontline stakeholders to adopt, make sense of, and routinize clinical practice.

Merging organisations, which was the favoured mechanism to integrate services by the government, were perceived as providing managers with the means to control the internal environments of their respective organisations so as to achieve integration goals. Several components of the functional dimension (Table 6) were viewed as partially or inadequately implemented. This suggest that despite the administrative mergers, additional efforts are needed at the local level to improve the implementation of health information systems, service management and human resources management. It will be important to dig in and understand the full dynamics of functional integration components such as health information systems, resources and services management in integrated care models.

The results of the second objective revealed several factors providers perceived as having a mild, moderate, or high potential influence on the implementation of integrated care models for older people (Tables 8, 9, 10, 11, 12). Most of these factors had been previously reported in the literature on implementation of integrated care [19,21,24,59], while some were new. It should be noted that, unlike England’s health system, where health care is provided by the National Health Service (NHS) and social care is provided by local governments, the Québec health system has combined health and social care since its inception in 1971. Hence, some factors commonly found in studies, such as co-location or integrated care trusts [26], intended to integrate health and social care organisations, were not salient in this study.

**Table 12:** Patient factors perceived as influencing the implementation of six dimensions of the Local Health Network for Older People.

Factors	Clinical dimension	Professional dimension	Organisational dimension	Systemic dimension	Functional dimension	Normative dimension

**Patients characteristics**	+	+			+	
**Family support**	++	++	++			
**Patient satisfaction**	+					
**Benefit to patients**	+		+	++		

Degree of influence: + mild influence; ++ moderate influence and +++ high influence.

The single most salient factor that providers perceived to have influenced the implementation of their integrated care model was government policy and funding support (Table 8). In fact, the three cases shared the same socio-economic and political context, marked notably by the merger of several establishments in 2004, which created the Health and Social Services Centres and empowered them with resources to create Local Health Networks for specific vulnerable populations in their territories. This support influenced the implementation of all dimensions of integration. For instance, government policy and funding facilitated the establishment of inter-organisational linkages and the implementation of administrative components of the Local Health Network, at the expense of clinical components, such as the case management function, which government has not made a priority to this date.

Our findings regarding the influence of merging organisations on the implementation of integrated care agree with those of Demers [60], who pointed out that mergers do not automatically lead to integrated practices because they can in fact negatively affect clinical practice. Nevertheless, the mergers seemed to create useful conditions even from the point of view of providers, for example by creating bridges with physicians. Several other factors that providers perceived as influencing the implementation of integrated care models in the structural, organisational, provider, innovation, and patient domains were consistent with those previously reported in integrated care literature [19,59,61]. Some factors that were not previously reported in implementation of integrated care studies included: use of the innovation by managers and policymakers, flexibility of the provider, willingness to work in semi-urban zones, cumbersomeness of the innovation, and lengthy duration of the innovation. Furthermore, these factors were consistent with several theoretical frameworks on the implementation of healthcare innovations [56,62,63], which suggests that they may influence the implementation of healthcare innovations in general [64], not just integrated care models. For instance, Damschroeder et al. [56] included adaptability, trialability, and complexity of the innovation in their model.

The authors recognise that these findings do not represent the only factors influencing the implementation of integrated care. A more complete picture will include the perspectives of other actors of implementation: policymakers, managers, and users (patients and caregivers). Recruiting providers from different units of the integrated care network (e.g. local community health centre, hospital, rehabilitation centre) strengthened this study by allowing triangulation of different perspectives across the continuum of care for older people. Most of the research participants were nurses and social workers, which may skew the results of this study towards their perspectives, causing information bias.

## Conclusion

The results reveal great similarities and moderate differences in the implementation of integrated care across the three cases, respectively showing the influence of the mandator of the innovation and the local context on the implementation of the integrated care model. Structural factors such as government policies and support, and organisational factors such as mergers positively influenced the implementation of systemic and organisational dimensions of integration at the expense of professional and clinical dimensions, reflecting the prioritisation of administrative components of integration over clinical components. Provider, innovation, and patient factors mildly or moderately influenced the implementation of the dimensions of integrated care. Stakeholders such as policymakers, managers, and providers should be informed concerning factors that they can strengthen to improve the routinisation and sustainability of similar integrated care models. This study also contributes to the scientific literature by proposing an innovative theoretical framework for analysing the implementation of integrated care and by identifying heretofore unreported factors that can influence the implementation of health care innovations. Hence, the present findings can guide future research on the implementation of health care innovations. Finally, it would be interesting to analyse the chronological process of implementing integrated care models.

## References

[1] Gröne, O and Garcia-Barbero, M. WHO European Office for Integrated Health Care Services: Integrated care: a position paper of the WHO European Office for Integrated Health Care Services. Int J Integr Care, 2001; 1: e21. 16896400 PMC1525335

[2] Shortell, SM, Gillies, RR, Anderson, DA, Mitchell, JB and Morgan, KL. Creating organized delivery systems: the barriers and facilitators. Journal of Healthcare Management, 1993; 38(4): 447–466. 10130607

[3] Leatt, P, Pink, GH and Guerriere, M. Towards a Canadian model of integrated healthcare. HealthcarePapers, 2000; 1(2): 13–35. DOI: 10.12927/hcpap..17216 12811063

[4] Contandriopoulos, AP, Denis, JL, Touati, N and Rodriguez, C. Groupe de recherche interdisciplinaire en santé: The integration of health care: dimensions and implementation. Montréal: GRIS, Université de Montréal; 2004.

[5] Evans, JM, Matheson, G, Buchman, S, MacKinnon, M, Meertens, E, Ross, J and Johal, H. Integrating cancer care beyond the hospital and across the cancer pathway: a patient-centred approach. Healthc Q., 2015; 17: 28–32. DOI: 10.12927/hcq.2014.24006 25562131

[6] Croghan, TW and Brown, JD. Integrating mental health treatment into the patient centered medical home Rockville, MD: Agency for Healthcare Research and Quality; 2010.

[7] Hébert, R, Durand, PJ, Dubuc, N and Tourigny, A. PRISMA Group: PRISMA: a new model of integrated service delivery for the frail older people in Canada. International Journal of Integrated Care, 2003; 3: e08 DOI: 10.5334/ijic.73 16896376 PMC1483944

[8] Lee, W, Eng, C, Fox, N and Etienne, M. PACE: a model for integrated care of frail older patients. Geriatrics, 1998; 53(6): 62–73. 9634107

[9] Kodner, DL and Kyriacou, CK. Fully integrated care for frail elderly: two American models. International Journal Of Integrated Care, 2000; 1(1): 1–19. DOI: 10.5334/ijic.11 PMC1533997 16902699

[10] Vedel, I, De Stampa, M, Bergman, H, Ankri, J, Cassou, B, Mauriat, C, Blanchard, F, Bagaragaza, E and Lapointe, L. A novel model of integrated care for the elderly: COPA, Coordination of Professional Care for the Elderly. Aging Clinical And Experimental Research, 2009; 21: 414–423. DOI: 10.1007/BF03327446 20154510

[11] Battersby, MW. Health reform through coordinated care: SA HealthPlus. BMJ: British Medical Journal, 2005; 330(7492): 662–665. DOI: 10.1136/bmj.330.7492.662 15775001 PMC554920

[12] Charles, L, Dobbs, B, Triscott, J and McKay, R. Care of the elderly program at the University of Alberta Meeting the challenges of treating the aging population. Canadian Family Physician, 2014; 60(11): e521–e526. 25551143 PMC4229174

[13] Hébert, R, Raîche, M, Dubois, M-F, Gueye, NDR, Dubuc, N, Tousignant, M and Group, P. Impact of PRISMA, a coordination-type integrated service delivery system for frail older people in Quebec (Canada): A quasi-experimental study. The Journals of Gerontology Series B: Psychological Sciences and Social Sciences, 2009; 65(1): 107–118. 10.1093/geronb/gbp027 19414866

[14] Poirier, LR, Descôteaux, S, Levesque, JF and Tourigny, A. Expedited knowledge synthesis on factors affecting implementation of integrated services networks for the elderly. INSPQ; 2015.

[15] Tourigny, A, Durand, PJ, Bonin, L, Hébert, R and Rochette, L. Quasi-experimental Study of the Effectiveness of an Integrated Service Delivery Network for the Frail Elderly. Canadian Journal on Aging, 2004; 23: 231–246. DOI: 10.1353/cja.2004.0038 15660297

[16] Beland, F, Bergman, H, Lebel, P and Dallaire, L. Integrated Services for Frail Elders (SIPA): A Trial of a Model for Canada. Canadian Journal on Aging, 2006; 25(1): 25–42. DOI: 10.1353/cja.2006.0019 16770746

[17] Kodner, DL and Spreeuwenberg, C. Integrated care: meaning, logic, applications, and implications – a discussion paper. International journal of integrated care, 2002; 2: 1–6. DOI: 10.5334/ijic.67 PMC1480401 16896389

[18] Kodner, D. All together now: a conceptual exploration of integrated care. Healthcare Quarterly (Toronto, Ont), 2008; 13: 6–15. 10.12927/hcq.2009.21091 20057243

[19] Mackie, S and Darvill, A. Factors enabling implementation of integrated health and social care: a systematic review. Br J Community Nurs, 2016; 21(2): 82–87. DOI: 10.12968/bjcn.2016.21.2.82 26844602

[20] Thiel, V, Sonola, L, Goodwin, N and Kodner, DL. Developing community resource teams in Pembrokeshire, Wales: Integration of health and social care in progress The King’s Fund, 2013; 1–29.

[21] Maruthappu, M, Hasan, A and Zeltner, T. Enablers and barriers in implementing integrated care. Health Systems & Reform, 2015; 1(4): 250–256. DOI: 10.1080/23288604.2015.1077301 31519094

[22] Le rôle des acteurs locaux, régionaux et ministériels dans l’intégration des services aux aînés en perte d’autonomie. [http://www.prismaquebec.ca/documents/document/fcrss_demerspdf.pdf].

[23] Coupe, M. Integrated care in Herefordshire: a case study. Journal of Integrated Care, 2013; 21(4): 198–207. DOI: 10.1108/JICA-01-2013-0001

[24] Ling, T, Brereton, L, Conklin, A, Newbould, J and Roland, M. Barriers and facilitators to integrating care: experiences from the English Integrated Care Pilots. International journal of integrated care, 2012; 12(129): 1–12. DOI: 10.5334/ijic.982 PMC3601528 23593044

[25] Syson, G and Bond, J. Integrating health and social care teams in Salford. Journal of Integrated Care, 2010; 18(2): 17–24. DOI: 10.5042/jic.2010.0132

[26] Challis, D, Stewart, K, Donnelly, M, Weiner, K and Hughes, J. Care management for older people: does integration make a difference? Journal of interprofessional care, 2006; 20(4): 335–348. DOI: 10.1080/13561820600727130 16905483

[27] Sheaff, R, Boaden, R, Sargent, P, Pickard, S, Gravelle, H, Parker, S and Roland, M. Impacts of case management for frail elderly people: a qualitative study. Journal of health services research & policy, 2009; 14(2): 88–95. DOI: 10.1258/jhsrp.2008.007142 19299262

[28] Fringer, A, Huth, M and Hantikainen, V. Nurses’ experiences with the implementation of the Kinaesthetics movement competence training into elderly nursing care: a qualitative focus group study. Scandinavian journal of caring sciences, 2014; 28(4): 757–766. DOI: 10.1111/scs.12108 24387733

[29] Christiani, Y, Byles, JE, Tavener, M and Dugdale, P. Exploring the implementation of poslansia, Indonesia’s community-based health programme for older people. Australas J Ageing, 2016; 35(3): E11–E16. DOI: 10.1111/ajag.12305 27198005

[30] Valentijn, PP, Boesveld, I, van der Klauw, D, Ruwaard, D, Struijs, J, Molema, J, Bruijnzeels, M and Vrijhoef, H. Towards a taxonomy for integrated care: a mixed-methods study. International Journal of Integrated Care, 2015; 15(1): e003 DOI: 10.5334/ijic.1513 25759607 PMC4353214

[31] Yin, R. Case Study Research: Design and Methods, 4 edn. Thousand Oaks, CA: Sage Publications Inc; 2003.

[32] Chaudoir, SR, Dugan, AG and Barr, CHI. Measuring factors affecting implementation of health innovations: a systematic review of structural, organizational, provider, patient, and innovation level measures. Implement Sci, 2013; 8(22): 1–20. DOI: 10.1186/1748-5908-8-22 23414420 PMC3598720

[33] Le bilan démographique du Québec: Édition 2016. [http://www.stat.gouv.qc.ca/statistiques/population-demographie/bilan2016.pdf].

[34] Turgeon, J, Deschênes, J-C and Simard, G. Le système de santé et de services sociaux In: La sécurité sociale au Québec: histoire et enjeux edn., Latullipe, D (ed.). 2016; 139–204. Québec: Les Presses de l’Université Laval.

[35] Couturier, Y, Belzile, L and Bonin, L. L’intégration des services en santé: une approche populationnelle Montréal: Les Presses de l’Université de Montréal 2016.

[36] Vedel, I, Monette, M, Beland, F, Monette, J and Bergman, H. Ten years of integrated care: backwards and forwards. The case of the province of Québec, Canada. International Journal of Integrated Care [serial online], 2011; 11(Special 10th Anniversary Edition): e004. 10.5334/ijic.574 PMC3111887 21677842

[37] Breton, M, Pineault, R, Levesque, J-F, Roberge, D, Da Silva, RB and Prud’homme, A. Reforming healthcare systems on a locally integrated basis: is there a potential for increasing collaborations in primary healthcare? BMC health services research, 2013; 13(1): 262 DOI: 10.1186/1472-6963-13-262 23835105 PMC3750424

[38] Breton, M, Denis, J-L and Lamothe, L. Incorporating public health more closely into local governance of health care delivery: lessons from the Québec experience. Canadian Journal of Public Health/Revue Canadienne de Santé Publique, 2010; 314–317. 21033545 10.1007/BF03405293 PMC6973886

[39] Breton, M, Haggerty, J, Roberge, D and Freeman, GK. Management continuity in local health networks. Int J Integr Care, 2012; 12: e14, 1–9. DOI: 10.5334/ijic.682 22977427 PMC3429137

[40] Outil de suivi de l’implantation des composantes du réseau de services intégrés Personnes âgées (OSIRSIPA). [https://www.google.ca/url?sa=t&rct=j&q=&esrc=s&source=web&cd=5&cad=rja&uact=8&ved=0ahUKEwjfnpv8m6_VAhUK4YMKHceuAigQFghKMAQ&url=https%3A%2F%2Fwww.excel-downloads.com%2Fattachments%2Fosirsipa-octobre-2010-v2-xlsx.244277%2F&usg=AFQjCNGTJNYdiNGuPSw-q53JXXvoq9xU3Q].

[41] Yin, R. Qualitative Research from Start to Finish, 2 New York: Guilford Publications; 2015.

[42] Sheridan, N, Kenealy, T, Kuluski, K, McKillop, A, Parsons, J, Wong-Cornall, C, Evans, JM, Grudniewicz, A, Gray, CS and Wodchis, WP. iCOACH. Implementing Integrated Care for Older Adults with Complex Health Needs. International Journal of Integrated Care, 2017; 17(Special collection): e12.

[43] Kuluski, K, Sheridan, N, Kenealy, T, Breton, M, McKillop, A, Shaw, J, Nie, JX, Upshur, RE, Baker, GR and Wodchis, WP. “On the Margins and Not the Mainstream:” Case Selection for the Implementation of Community based Primary Health Care in Canada and New Zealand. International journal of integrated care, 2017; 17(2). DOI: 10.5334/ijic.2501 PMC5624111 28970756

[44] Breton, M, Grey, CS, Sheridan, N, Shaw, J, Parsons, J, Wankah, P, Kenealy, T, Baker, R, Belzile, L and Couturier, Y. Implementing Community Based Primary Healthcare for Older Adults with Complex Needs in Quebec, Ontario and New-Zealand: Describing Nine Cases. Int J Integr Care, 2017; 17(2): 1–14. DOI: 10.5334/ijic.2506 PMC5624082 28970753

[45] Fusch, PI and Ness, LR. Are we there yet? Data saturation in qualitative research. The Qualitative Report, 2015; 20(9): 1408–1416.

[46] NVIVO: THE #1 SOFTWARE FOR QUALITATIVE DATA ANALYSIS. [http://www.qsrinternational.com/nvivo-product/nvivo11-for-windows].

[47] Campbell, JL, Quincy, C, Osserman, J and Pedersen, OK. Coding in-depth semistructured interviews: Problems of unitization and intercoder reliability and agreement. Sociological Methods & Research, 2013; 42(3): 294–320. DOI: 10.1177/0049124113500475

[48] Finlay, L. ‘Rigour’, ‘ethical integrity’ or ‘artistry’? Reflexively reviewing criteria for evaluating qualitative research. British Journal of Occupational Therapy, 2006; 69(7): 319–326. DOI: 10.1177/030802260606900704

[49] Dixon-Woods, M, Agarwal, S, Jones, D, Young, B and Sutton, A. Synthesising qualitative and quantitative evidence: a review of possible methods. Journal of health services research & policy, 2005; 10(1): 45–53. DOI: 10.1177/135581960501000110 15667704

[50] Marcus, AA. Implementing externally induced innovations: A comparison of rule-bound and autonomous approaches. Academy of management journal, 1988; 31(2): 235–256. DOI: 10.2307/256547

[51] Helfrich, CD, Savitz, LA, Swiger, KD and Weiner, BJ. Adoption and implementation of mandated diabetes registries by community health centers. American journal of preventive medicine, 2007; 33(1): S50–S65. DOI: 10.1016/j.amepre.2007.04.002 17584591

[52] Barry, MJ and Edgman-Levitan, S. Shared decision making — the pinnacle of patient-centered care. New England Journal of Medicine, 2012; 366(9): 780–781. DOI: 10.1056/NEJMp1109283 22375967

[53] Ouwens, M, Wollersheim, H, Hermens, R, Hulscher, M and Grol, R. Integrated care programmes for chronically ill patients: a review of systematic reviews. International journal for quality in health care, 2005; 17(2): 141–146. DOI: 10.1093/intqhc/mzi016 15665066

[54] Béland, F and Hollander, MJ. Integrated models of care delivery for the frail elderly: international perspectives. Gac Sanit, 2011; 25(Suppl 2): 138–146. DOI: 10.1016/j.gaceta.2011.09.003 22088903

[55] Wodchis, W, Dixon, A, Anderson, G and Goodwin, N. Integrating care for older people with complex needs: key insights and lessons from a seven-country cross-case analysis. International Journal of Integrated Care, 2015; 15(6): None. 10.5334/ijic.2249 PMC4628509 26528096

[56] Damschroder, LJ, Aron, DC, Keith, RE, Kirsh, SR, Alexander, JA and Lowery, JC. Fostering implementation of health services research findings into practice: a consolidated framework for advancing implementation science. Implement Sci, 2009; 4(50): 1–15. DOI: 10.1186/1748-5908-4-50 19664226 PMC2736161

[57] Ayuso, S, Ángel Rodríguez, M, García-Castro, R and Ángel Ariño, M. Does stakeholder engagement promote sustainable innovation orientation? Industrial Management & Data Systems, 2011; 111(9): 1399–1417. DOI: 10.1108/02635571111182764

[58] Huber, GP. Organizational learning: The contributing processes and the literatures. Organization science, 1991; 2(1): 88–115. DOI: 10.1287/orsc.2.1.88

[59] Robertson, H. Integration of health and social care. A review of literature and models Implications for Scotland In: Royal College of Nursing Scotland, 2011; 1–42.

[60] Demers, L. Mergers and integrated care: the Quebec experience. International journal of integrated care, 2013; 13(1): 1–4. DOI: 10.5334/ijic.1140 PMC3653283 23687474

[61] Nolte, E, Frølich, A, Hildebrandt, H, Pimperl, A, Schulpen, GJ and Vrijhoef, HJ. Implementing integrated care: A synthesis of experiences in three European countries. International Journal of Care Coordination, 2016; 19(1–2): 5–19. DOI: 10.1177/2053434516655626

[62] Rogers, EM. Diffusion of innovations, 5th edn New York: Simon and Schuster; 2003.

[63] Greenhalgh, T, Robert, G, Macfarlane, F, Bate, P and Kyriakidou, O. Diffusion of innovations in service organizations: systematic review and recommendations. Milbank Quarterly, 2004; 82(4): 581–629. DOI: 10.1111/j.0887-378X.2004.00325.x 15595944 PMC2690184

[64] Huijg, JM, Gebhardt, WA, Verheijden, MW, van der Zouwe, N, de Vries, JD, Middelkoop, BJ and Crone, MR. Factors influencing primary health care professionals’ physical activity promotion behaviors: a systematic review. International journal of behavioral medicine, 2015; 22(1): 32–50. DOI: 10.1007/s12529-014-9398-2 24788314

